# Effects of individualized comprehensive nutritional support on inflammatory markers, serum amylase (AMS), prealbumin (PA), albumin (ALB), calcium ion (Ca2+) in patients with severe pancreatitis

**DOI:** 10.5937/jomb0-48944

**Published:** 2024-11-16

**Authors:** Xiaoxi Liu, Wei Meng

**Affiliations:** 1 Changchun Humanities and Sciences College, School of Nursing and Well-being, Changchun, China

**Keywords:** severe acute pancreatitis, individualized comprehensive nutrition support, inflammatory index, biochemical index, vegetative state, teški akutni pankreatitis, individualizovana sveobuhvatna nutricionistička podrška, inflamatorni indeks, biohemijski indeks, vegetativno stanje

## Abstract

**Background:**

The aim of the paper was to study the effectiveness of individualized comprehensive nutritional support on inflammatory markers, serum amylase (AMS), prealbumin (PA), albumin (ALB), calcium ion (Ca2+) in patients with severe acute pancreatitis (SAP).

**Methods:**

102 participants with SAP treated in our hospital were chosen as the study objects. The participants were randomly split into a control group and an observation group and both groups were given routine treatment. Additionally, the observation group received individualized comprehensive nutrition support. The inflammatory indexes, biochemical indexes and symptom improvement time were observed and analyzed on the day of admission, intervention 1d, intervention 3d, intervention 7d and intervention 14d.

**Results:**

Among the 102 patients included in this study, 3 cases had sudden exacerbation during the intervention, 1 case had clinical data missing >10%, and 1 case voluntarily withdrew due to personal factors, all of which were eliminated. Finally, the effective data for 97 patients were recovered. There were significant differences in the hypersensitive C-reactive protein (hs-CRP), white blood cell count (WBC), procalcitonin (PCT) and interleukin1b (IL-1β) between the two groups. The inter-group, time and interaction differences of AMS, PA, ALB and Ca2+ levels were significantly different. The recovery time of abdominal pain, bowel sound, blood amylase level and urine amylase level in the observation group was inferior to the control group, and the differences were statistically significant (P<0.05).

**Conclusions:**

Individualized comprehensive nutritional support is more conducive to the improvement of inflammatory indexes in SAP patients and can accelerate symptom relief and promote the recovery of nutritional status.

## Abstract (sr)

**Uvod:**

Cilj rada bio je da se ispita efikasnost individualizovane sveobuhvatne nutricionističke podrške na inflamatorne markere, serumsku amilazu (AMS), prealbumin (PA), albumin (ALB) i jon kalcijuma (Ca2+) kod pacijenata sa teškim akutnim pankreatitisom (SAP).

**Metode:**

Za potrebe studije izabrana su 102 učesnika sa teškim akutnim pankreatitisom (SAP) lečena u našoj bolnici. Učesnici su nasumično podeljeni u kontrolnu grupu i posmatranu grupu, pri čemu su obe grupe dobijale rutinsku terapiju. Posmatrana grupa je dodatno dobijala individualizovanu sveobuhvatnu nutricionističku podršku. Inflamatorni i biohemijski indeksi, kao i vreme poboljšanja simptoma, posmatrani su i analizirani na dan prijema, nakon 1 dana intervencije, 3 dana intervencije, 7 dana intervencije i 14 dana intervencije.

**Rezultati:**

Među 102 pacijenta uključenih u ovu studiju, kod 3 učesnika studije je došlo do naglog pogoršanja tokom intervencije, kod jednog slučaja su klinički podaci nedostajali više od 10%, a jedan učesnik studije je dobrovoljno odustao zbog ličnih razloga, te su svi oni isključeni iz studije. Na kraju su validni podaci prikupljeni za 97 pacijenata. Postojale su značajne razlike u visokoosetljivom C-reaktivnom proteinu (hs-CRP), broju belih krvnih zrnaca (WBC), prokalcitoninu (PCT) i interleukinu 1b (IL-1b) između dve grupe. Razlike među grupama, vremenske razlike i interakcije nivoa AMS, PA, ALB i Ca2+ su bile značajne. Vreme oporavka od bolova u stomaku, zvukova u crevima, nivoa amilaze u krvi i nivoa amilaze u urinu u posmatračkoj grupi bilo je kraće u odnosu na kontrolnu grupu, a razlike su bile statistički značajne (P<0,05).

**Zaključak:**

Individualizovana sveobuhvatna nutricionistička podrška je pogodnija za poboljšanje inflamatornih indeksa kod pacijenata sa teškim akutnim pankreatitisom (SAP), i može da ubrza olakšanje simptoma i podstakne oporavak nutritivnog statusa.

## Introduction

Severe acute pancreatitis (SAP) is a special type of acute pancreatitis with a mortality rate of 20% to 30% [1]. With the continuous development of medical technology, the diagnosis and treatment level of severe acute pancreatitis has been improved, which has significantly improved the clinical efficacy of the disease. However, since most of the disease is acute, it can enhance the patient’s glycoconeogenesis response. In addition, the body needs to expend a lot of energy during the recovery period, making patients prone to malnutrition. This situation is not only detrimental to the patient’s disease remission and physical rehabilitation but also the decreased immune function caused by nutritional deficiency will increase the infection rate of the patient, thus further increasing the risk of death [2]. Therefore, strengthening the nutritional support during the treatment of SAP patients has a positive significance for maintaining the nutritional level of the body and stimulating the physical recovery of patients. At present, there are many types of nutritional support for SAP patients. However, clinical studies have pointed out that although nutritional support contributes to the improvement of nutritional status, reduces the damage to organs, protects intestinal barrier function, and promotes wound healing, there is still controversy over the choice of nutritional support [3] [4]. The SAP is complex, and the symptoms of different patients are also different, which makes it difficult to achieve a good prognosis for all patients with conventional nutritional support alone. Individualized nutrition support can develop targeted nutrition management programs according to patients’ specific conditions to fully meet their nutritional needs, thus contributing to enhancing their nutritional status and reducing adverse clinical outcomes [5] [6] [7] [8] [9]. Therefore, to seek a nutrition support program that is more suitable for SAP patients, this study implemented individualized comprehensive nutrition support strategies for SAP patients and studied and analyzed its application effects, as reported below.

## Materials and methods

### Subjects

A total of 139 SAP sufferers were admitted to our hospital during 2022.8～2023.12. According to the formula [n=2σ^2^(Z_α_+Z_β_)÷*β* ^2^] [10], the sample size of this study should be no less than 102 cases. Therefore, 102 SAP patients in our hospital were randomly chosen as research objects.

Inclusion criteria: ① SAP related diagnostic criteria in Chinese Diagnostic Guidelines for Acute pancreatitis [10] [11]; ② Age ≥18 years old; ③ The time from onset to admission was less than 48h; ④ Acute Physiology and Chronic Health II (APACHE II) [12] score >8.

Exclusion criteria: ① Combined with congenital immune disease; ② Combined with malignant tumour; ③ Complicated with severe hepatic and renal dysfunction; ④ Sepsis secondary to pancreatic infection; ⑤ Combined with intestinal motility disorders; ⑥ Complicated with intestinal obstruction requiring surgical treatment; ⑦ There is a disorder of consciousness, or combined with mental illness.

Shedding criteria: ① Sudden deterioration of the condition during treatment; ② Active withdrawal due to various reasons during treatment; ③ Data collection missing >10%.

The Medical Ethics Committee approved this paper in accordance with the Declaration of Helsinki [13].

### Methods

The two groups were given routine treatment, including fluid resuscitation and anti-infection therapy. Patients in the Control Group (CG) received routine nutrition support, and patients in the Observation Group (OG) received individualized comprehensive nutrition support. For 14 days, Nutritional Support programs in the study were designed according to Nutritional Support in Patients with Severe Acute Pancreatitis-Current Standards [14].

### Routine nutritional support

Parenteral nutrition support was given, and the nutrient solution was injected after the central or peripheral veins were opened. Nutrient solution formula: amino acid 1.3 g/(kg·d), heat nitrogen ratio 150 kcal:1g, sugar to lipid ratio 1:1, an appropriate amount of trace elements, vitamins, etc.

### Individualized comprehensive nutrition support

(1) Set up a team: set up an individualized nutrition support team, including 1 head nurse, 1 attending physician, 3 dietitians and 5 specialist nurses. After the team is formed, the head nurse will organize the team members to learn the relevant theoretical knowledge and operational process of individualized nutrition support. After the study, the business assessment will be conducted, and all the team members are required to be familiar with the relevant knowledge. (2) Make a plan: The team will conduct a comprehensive assessment of the nutritional status and body function status of patients under the leadership of attending physicians and nutritionists and rationally adopt NRS2002 or other nutritional status screening tools. According to the evaluation results and the current condition of the patient, the nutritional needs of the patient were determined, and the future nutritional status and needs were predicted. And finally, the individualized comprehensive nutrition program was determined. (3) Implementation plan: The nutrition support program consists of three stages: total parenteral nutrition support parenteral + enteral nutrition support total enteral nutrition support.

① Total parenteral nutrition support stage: The patient was given lipeptide (national drug approvalnumber H20045404, Fuzhou Haiwangfu Pharmaceutical Co., LTD., size 100 mL:20 g) 100 mL/d and 500 mL normal saline intravenous input; However, the maximum daily input dose of peptides should not exceed 2.0 mL/kg for 5 days.

② Parenteral + enteral nutrition support stage: After the gastrointestinal function of the patient recovers, based on parenteral support, the patient can be fully injected through a spiral naso-intestinal tube (National drug approval number H20030012, Nuditia Pharmaceutical Co., LTD., specification 0.75 kcal/mL); The initial dose was 200 kcal/d, and the degree of enteral nutrition tolerance was evaluated by the enteral nutrition tolerance scale every day. If the score was 0 to 6, enteral nutrition was continued, and the dose was gradually adjusted to 1800 kcal/d. Scores 7–12 continued enteral nutrition but slowed down the input speed; Enteral nutrition was stopped for 5 days with scores ≥13.

③ Total enteral nutrition support: parenteral nutrition was discontinued, and the nutritional intake of patients was provided by enteral nutrition. The total dose was 1800 kcal/d through a spiral naso-intestinal tube. Nutrient solution formula: Each 500 kcal solution contains 20 g protein, 19.5 g fat, 61.5 g carbohydrate, 7.5 g dietary fibre, 2.5 g mineral and 150 IU vitamin; Gastric residual volume was measured daily. Gastric residual volume >200 mL, maintain the original nutrient solution input speed; Gastric residual volume between 100~200 mL, slow down the input speed; Stomach residual volume <100 mL, increase input speed, lasting for 4d.

④ Nutritional supplement: After more than 7 days of nutritional support, fasting venous blood of patients in the morning was collected to detect nutritional levels. If the patient’s haemoglobin is <80 g/L, the appropriate amount of amino acids, potassium, protein and other nutrients should be added to the patient.

### Observational indicators and data collection methods

5 mL of fasting venous blood was collected on the first day of admission, intervention 1d, intervention 3d, intervention 7d and intervention 14d, respectively, and was stored for examination after centrifugation.

### Inflammatory indicators

Hypersensitive C-reactive protein (hs-CRP) and interleukin-1β(IL-1β) were decided by Enzyme-Linked Immunosorbent Assay (ELIA), procalcitonin (PCT) was decided by enzyme-linked immunofluorescence assay, and white blood cell count (WBC) was determined by blood analyzer.

### Biochemical indicators

Serum amylase (AMS) and albumin (ALB) were determined by ELIA. Prealbumin (PA) was detected by projective turbidimetry. Calcium ion (Ca^2+^) was determined by the GBHA method.

### Symptom improvement time

The time of abdominal pain relief, bowel sound recovery, AMS level in blood and AMS level in urine were recorded.

### Statistical methods

SPSS 26.0 was applied to analyze the information. This paper’s data is expressed as mean ± standard deviation (x̄±s). Independent *t*-test was performed between groups. GraphPad Prism 9 was used to draw the line chart of index changes. The number of use cases and percentage [n (%)] of the counting data were expressed, and *χ* ^2^ test was performed. *P*<0.05 was considered statistically significant.

## Results

### Comparison of baseline data

Among the 102 patients included in this study, 3 cases were suddenly aggravated during the intervention, 1 case had clinical data missing >10%, and 1 case voluntarily withdrew due to personal factors, all of which were eliminated. Finally, the effective data of 97 patients were recovered, including 48 cases in the CG and 49 cases in the OG. The effective data recovery rate was 95.10%. In Table 1, there were no statistically significant differences in gender, age, bodymass index (BMI), APACHE II score, nutritional risk screening 2002 (NRS2002), time from onset to admission, serum lipase (LPS), pathological types (*P*<0.05).

**Table 1:** Comparison of baseline data [n(%),(x̄±s)]. Note: BMI Body Mass Index, APACHEII Acute Physiological and Chronic Health Score II, NRS2002 Nutritional Risk Screening 2002, LPS serum lipase

Item		CG (n=48)	OG (*n*=49)	*χ^2^/t*	*P*
Gender	Male	25(52.08)	27(55.10)	0.089	0.766
	Female	23(47.92)	22(44.90)		
Age (years)		41.25±5.48	40.63±4.85	0.588	0.558
BMI (kg/m^2^)		22.68±2.22	23.00±2.34	-0.692	0.491
APACHE II Score (points)		16.44±3.00	15.33±4.17	1.503	0.136
NRS2002 Score (points)		0.94±0.43	0.98±0.43	-0.479	0.633
Time from onset to admission (h)		7.94±1.80	7.39±2.08	1.390	0.168
LPS (U/L)		847.67±46.77	853.43±45.49	-0.615	0.540
Disease type	Biliary pancreatitis	18(37.50)	17(34.69)	0.757	0.860
	Idiopathic pancreatitis	15(31.25)	14(28.57)		
	Alcoholic pancreatitis	13(27.08)	14(28.57)		
	Hyperlipidemic pancreatitis	2(4.17)	4(8.16)		

### Comparison of inflammatory indexes between the two groups

**Figure 1:**
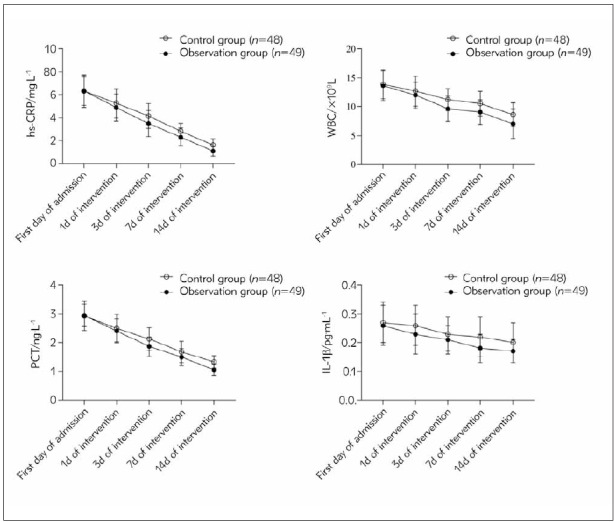
Line chart of inflammatory indexes between the two groups.

In Table 2 and Figure 1, intergroup, time and interaction differences in hs-CRP, WBC, PCT and IL-1β levels were statistically significant (*P*<0.05). The intra-group comparison showed that the levels of hs-CRP, WBC, PCT and IL-1β in the two groups showed a decreasing trend on the intervention day 14, and the levels of hs-CRP, WBC, PCT and IL-1β on the intervention day 14 were inferior to the first day of admission, intervention day 1, intervention day 3 and intervention day 7 (*P*<0.05). There was no remarkable discrepancy in hs-CRP, WBC, PCT and IL-1β levels on the first day of admission and the intervention day 1 (*P*>0.05). The hs-CRP, WBC, PCT and IL-1β levels in OG were significantly lower than those in CG at 3d, 7d and 14d after intervention (*P*<0.05).

**Table 2:** Comparison of inflammatory indexes (x̄±s). Note: hs-CRP hypersensitive C-reactive protein, WBC white blood cell count, PCT procalcitonin, IL-1βinterleukin1β<br>hs-CRP: F _inter-group_/F _time_ /F _interaction_=4.443/1384.504/34.612，Pinter-group/P _time_/P _interaction_=0.038/<0.001/<0.001<br>WBC:F _inter-group_/F _time _/F _interaction_=5.823/3062.035/37.565，Pinter-group/P _time _/P _interaction_=0.018/<0.001/<0.001<br>PCT: F _inter-group_/F _time_/F _interaction_=4.838/4174.247/83.321，Pinter-group/P _time _/P_interaction_=0.030/<0.001/<0.001<br>IL-1β: F _inter-group_/F _time_/F _interaction_=4.185/466.709/25.002，Pinter-group/P _time _/P _interaction_=0.044/<0.001/<0.001

Item		CG (*n*=48)	OG (*n*=49)	*t*	*P*
hs-CRP (mg/L)	First day of admission	6.32±1.42	6.33±1.26	-0.003	0.998
	1d	5.25±1.26	4.88±1.18	1.513	0.134
	3d	4.15±1.07	3.50±1.13	2.881	0.005
	7d	2.84±0.67	2.33±0.77	3.529	0.001
	14d	1.64±0.54	1.10±0.44	5.323	<0.001
WBC (×10^9^/L)	First day of admission	13.86±2.54	13.62±2.60	0.468	0.641
	1d	12.69±2.61	11.98±2.26	1.426	0.157
	3d	11.19±1.92	9.61±2.19	3.768	<0.001
	7d	10.51±2.13	9.04±2.10	3.422	0.001
	14d	8.62±2.03	7.04±2.54	3.368	0.001
PCT (ng/L)	First day of admission	2.94±0.51	2.95±0.38	-0.106	0.916
	1d	2.51±0.48	2.41±0.42	1.132	0.261
	3d	2.13±0.40	1.86±0.35	3.498	0.001
	7d	1.67±0.37	1.49±0.29	2.643	0.010
	14d	1.32±0.21	1.05±0.20	6.337	<0.001
IL-1β (pg/mL)	First day of admission	0.27±0.07	0.26±0.07	0.859	0.393
	1d	0.26±0.07	0.23±0.07	1.792	0.076
	3d	0.23±0.06	0.21±0.05	2.001	0.048
	7d	0.22±0.07	0.18±0.05	3.145	0.002
	14d	0.20±0.07	0.17±0.04	2.538	0.013

### Comparison of biochemical indexes between the two groups

**Figure 2:**
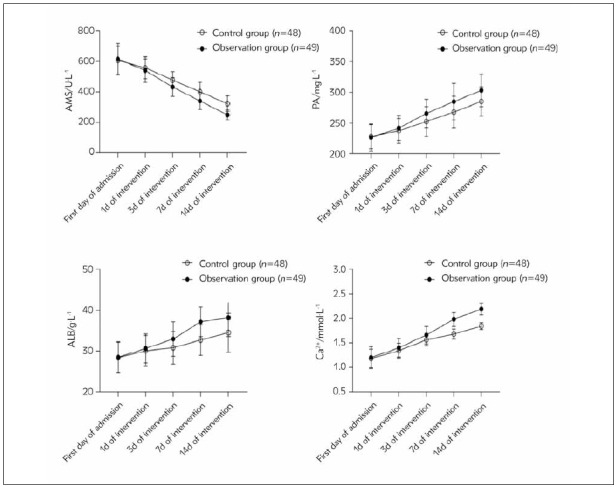
Line chart of biochemical index changes between the two groups.

In Table 3 and Figure 2, intergroup, time and interaction differences in AMS, PA, ALB and Ca^2+^ levels had statistical significance (*P*<0.05). The intragroup comparison showed that the AMS level of patients in both groups showed a decreasing trend from the first day of admission to 14 days of intervention. The PA, ALB and Ca^2+^ levels all showed an increasing trend from the first day of admission to 14 days of intervention. Moreover, the AMS level of the two groups on intervention day 14 was lower than that on the first day, intervention day 1, intervention day 3 and intervention day 7. The PA, ALB and Ca^2+^ levels on intervention day 14 were higher than those on the first day, intervention day 1, intervention day 3 and intervention day 7. The above differences had statistical significance (*P*<0.05). There was no significant difference in AMS, PA, ALB and Ca^2+^ levels between the two groups on the first day and 1 day after intervention (*P*>0.05). The AMS levels in the OG were significantly inferior to CG at 3d, 7d and 14d. The PA, ALB and Ca^2+^ levels were higher than those of CG (*P*<0.05).

**Table 3:** Comparison of inflammatory indexes (x̄±s). Note: AMS amylase, PA prealbumin, ALB albumin, Ca^2+^ calcium ion<br>AMS *F*_inter-group_ /*F*_time _/*F*_interaction_=7.989/2575.696/30.459 *P*_inter-group_/*P*_time_/*P*_interaction_=0.006/<0.001/<0.001<br>PA *F*_inter-group_ /*F*_time _/*F*_interaction_=4.655/2365.480/64.766 *P*_inter-group_/*P*_time_/*P*_interaction_=0.033/<0.001/<0.001<br>ALB *F*_inter-group_ /*F*_time _/*F*_interaction_=7.572/2494.350/221.279 *P*_inter-group_/*P*_time_/*P*_interaction_=0.007/<0.001/<0.001<br>Ca^2+^ *F*_inter-group_/*F*_time _/*F*_interaction_=30.401/1409.715/210.336 *P*_inter-group_/*P*_time_/*P*_interaction_=<0.001/<0.001/<0.001

Item		CG (*n*=48)	OG (*n*=49)	*t*	*P*
AMS (U/L)	First day of admission	606.52±94.64	615.84±101.72	-0.467	0.642
	1d	558.16±71.93	540.22±76.23	1.192	0.236
	3d	477.72±54.33	432.18±61.83	3.850	<0.001
	7d	401.22±62.79	341.01±55.73	4.997	<0.001
	14d	322.99±50.73	248.85±32.58	8.582	<0.001
PA (mg/L)	First day of admission	227.98±19.62	226.38±22.16	0.376	0.707
	1d	237.50±19.97	241.97±20.48	-1.088	0.279
	3d	252.54±24.34	265.52±23.34	-2.683	0.009
	7d	267.80±25.74	284.95±29.85	-3.028	0.003
	14d	285.28±23.60	303.31±26.47	-3.539	0.001
ALB (g/L)	First day of admission	28.36±3.80	28.49±3.74	-0.174	0.862
	1d	30.05±3.75	30.63±3.53	-0.786	0.434
	3d	30.80±4.00	32.93±4.21	-2.555	0.012
	7d	32.69±3.74	37.17±3.61	-6.012	<0.001
	14d	34.51±4.76	38.16±4.60	-3.848	<0.001
Ca^2+^ (mmol/L)	First day of admission	1.18±0.19	1.20±0.22	-0.517	0.606
	1d	1.34±0.15	1.40±0.19	-1.596	0.114
	3d	1.56±0.12	1.66±0.18	-3.352	0.001
	7d	1.68±0.10	1.98±0.14	-11.995	<0.001
	14d	1.84±0.07	2.19±0.12	-18.171	<0.001

### Comparison of clinical symptom improvement time of the two groups

In Table 4 and Figure 3, the improvement time of clinical symptoms in the OG was inferior to CG, with statistical significance (*P*<0.05).

**Table 4:** Comparison of clinical symptom improvement time (x̄±s, d).

Item	CG (*n*=48)	OG (*n*=49)	*t*	*P*
Abdominal pain relief	6.00±0.97	4.59±0.91	7.381	<0.001
Bowel sounds recovered	4.85±0.71	3.78±0.69	7.591	<0.001
Blood amylase levels returned to normal	6.88±0.87	5.65±0.88	6.895	<0.001
Urine amylase levels returned to normal	7.00±0.77	5.82±0.67	8.087	<0.001

## Discussion

Acute Pancreatitis (AP) is a common disease of the digestive system. The main mechanism of AP is the activation of pancreatic enzymes in the pancreas, which causes digestion, edema, bleeding and even necrosis of pancreatic tissue. Compared with mild AP, SAP is more difficult to treat. In addition, most patients are complicated with organ failure, which can endanger the life of patients in severe cases [15] [16]. Existing studies have pointed out [17] that most SAP patients are in a hypercatabolic state after the disease. Its energy consumption is large, and the clinical need to maintain the body’s nutritional state through nutritional support to avoid patients due to the decline of immune function while affecting treatment progress. In this study, individualized comprehensive nutrition support was implemented for SAP patients. By setting up a nutrition support team and combining nutritional status and physical function, an individualized nutrition support program was developed to meet patients’ individual nutritional needs. Compared with conventional nutritional support, individualized comprehensive nutritional support is more targeted and more conducive to supplementing patients with nutritional deficiencies. In addition, various nutritional support evaluation tools are flexibly used to optimize the nutritional support process to accelerate the treatment progress, reduce complications, and promote the recovery of patients.

The paper showed that the intergroup, time and interaction differences of hs-CRP, WBC, PCT and IL-1β levels had statistical significance (*P*<0.05). Individualized comprehensive nutrition support program was more conducive to the improvement of verification indicators than conventional nutrition support, which was consistent with the results of Sun et al. [18] and Chen et al. [19] hs-CRP is a sensitive indicator used to distinguish low-level inflammatory states in clinical practice [20], while PCT is a specific indicator secreted by thyroid C cells in normal circumstances to evaluate the systemic inflammatory response of the body [21]. hs-CRP and PCT levels are generally low in healthy people, but when the body has a severe infection, it can lead to increased levels of hs-CRP, PCT and other related inflammatory factors. Previous studies have also pointed out that hs-CRP can better reflect the degree of necrosis of pancreatic cells in patients with AP, while PCT is more conducive to judging the severity of early disease in patients with AP than other inflammatory factors [22] [23]. WBC is an important part of the body’s defence system, and domestic and foreign studies [24] [25] have proved that WBC is one of the important indicators for predicting the severity of AP patients. IL-1β plays an important role in mediating infection, trauma and inflammatory response in the body [26]. Regarding the influence of nutritional support on the level of inflammatory factors, Qiu et al. [27] pointed out that SAP would cause damage to the gastrointestinal barrier and activate macrophages, thus accelerating the release of inflammatory mediators and aggravating the disease. This study used a combination of enteral and parenteral nutrition support. Among them, parenteral nutrition support provides a good nutritional supplement for patients with poor gastrointestinal tolerance. The progressive enteral nutrition support can not only prevent the occurrence of intestinal mucosal villi atrophy caused by long-term parenteral nutrition support, avoid damage to the intestinal mucosal barrier, ensure that the patient’s pancreas gets enough rest, but also realize the direct contact between the intestinal mucosa and the nutrient substrate, meet the nutritional requirements of the intestinal mucosa, and improve the intestinal mucosal blood circulation. This different distribution of enteral and parenteral nutritional support allows patients to maximize the effect of nutritional support. In addition, early enteral nutrition is a hydrolyzed digestive nutrient, which can be rapidly absorbed by intestinal epithelial cells, promote the restoration of positive nitrogen balance, accelerate wound healing, and inhibit the release of inflammatory factors such as hs-CRP and PCT [28].

The intergroup, time and interaction differences of AMS, PA, ALB and Ca^2+^ levels had statistical significance (*P*<0.05). The results indicated that individualized comprehensive nutritional support was more conducive to the improvement of AMS, PA, ALB and Ca^2+^ levels in SAP patients, which was consistent with the results of Li et al. [29] and Garg et al. [30]. This is consistent with recent studies that have shown the importance of nutritional support in managing inflammatory markers and improving clinical outcomes in patients with severe illnesses [14] [31]. Accordingly, a study found that patients with acute pancreatitis and serum albumin levels less than 25 g/L anytime during hospitalization had a 16.8-fold higher risk of death and 48.8-fold higher risk of severe acute pancreatitis than patients with normal albumin levels [32]. This suggests that maintaining albumin levels through nutritional support could significantly improve patient outcomes.

Moreover, our study found significant intergroup, time, and interaction differences in AMS levels. This is consistent with a study exploring the prevalence and clinical characteristics of elevated pancreatic enzymes (amylase and lipase) and its association with acute pancreatitis in patients with severe fever and thrombocytopenia syndrome [33].

AMS, one of the main types of amylases in serum, is a glycoside chain hydrolase derived fromthe pancreas and is currently an important indicator for diagnosing AP. After the SAP pancreas is damaged, a large amount of pancreatic amylase will be secreted, increasing the amylase content absorbed by the blood and increasing AMS levels. However, in clinical practice, large areas of pancreatic tissue necrosis caused by AP may also occur, resulting in an abnormal increase in AMS levels in the body, which makes it impossible to assess the disease accurately. Therefore, AMS should not be used as the only diagnostic indicator in the clinical diagnosis of SAP, and it should be combined with other indicators for comprehensive evaluation [34]. PA and ALB can reflect themetabolic status of the liver. When the liver function is damaged or under stress, PA and ALB can reflect the level of decomposition and anabolism of the body to a certain extent. When the body PA and ALB are low, it indicates that the patient is malnourished. As a second messenger responsible for intracellular signalling to trigger physiological changes, serum Ca^2+^ can promote the exocytosis of digestive enzymes. With the continuous development of AP, the destruction of pancreatic cells may cause fatty acids to flow into the abdominal cavity or decompose fats to produce excessive fatty acids, which combine with Ca^2+^ in the body to form fatty acid calcium, resulting in a large amount of Ca^2+^ consumption in the body and a decline in the level of Ca^2+^ in the body [35]. Individualized comprehensive nutrition support is combined with the patient’s physical state to carry out accurate nutritional needs assessment, meet the different nutritional needs of patients with different clinical manifestations, and stimulate the physical recovery of patients. After pancreatic tissue repair, pancreatic function is gradually restored, which reduces the release of pancreatic amylase and consumption of Ca^2+^ in the body. The level of AMS decreased, and the level of Ca^2+^ increased. While the nutritional status of patients continues to improve, thelevels of PA and ALB in patients will gradually become normal. Another study in this study showed that the recovery time of abdominal pain, intestinal ringing sound, and blood and urine amylase levels in the OG were inferior to CG, with statistical significance (*P*<0.05). These results suggest that individualized comprehensive nutritional support can accelerate the recovery of patients. The results also confirm that individualized comprehensive nutritional support can more quickly restore the inflammatory markers and related biochemical markers to normal levels in SAP patients.

## Conclusion

Our study demonstrated that individualized comprehensive nutritional support significantly improved inflammatory markers and biochemical indexes in patients with severe acute pancreatitis. Furthermore, it expedited the recovery of abdominal pain, bowel sound, and amylase levels, underscoring the potential of nutritional support in enhancing patient outcomes.

## Dodatak

### Conflict of interest statement

All the authors declare that they have no conflict of interest in this work.
